# Amplitude Integrated Electroencephalogram as a Prognostic Tool in Neonates with Hypoxic-Ischemic Encephalopathy: A Systematic Review

**DOI:** 10.1371/journal.pone.0165744

**Published:** 2016-11-01

**Authors:** Ruth del Río, Carlos Ochoa, Ana Alarcon, Juan Arnáez, Dorotea Blanco, Alfredo García-Alix

**Affiliations:** 1 Department of Neonatology, Institut de Recerca Pediàtrica Hospital Sant Joan de Déu, Barcelona, Spain; 2 Research Unit, Hospital Virgen de la Concha, Zamora, Spain; 3 Department of Neonatology, Oxford University Hospitals NHS Foundation Trust, Oxford, United Kingdom; 4 Department of Neonatology, Hospital Universitario Burgos, Burgos, Spain; 5 Department of Neonatology, Hospital Universitario Gregorio Marañón, Madrid, Spain; Centre Hospitalier Universitaire Vaudois, FRANCE

## Abstract

**Introduction:**

Perinatal management and prognostic value of clinical evaluation and diagnostic tools have changed with the generalization of therapeutic hypothermia (TH) in infants with hypoxic-ischemic encephalopathy (HIE)

**Aim:**

to ascertain the prognostic value of amplitude integrated electroencephalogram (aEEG) in neonates with HIE considering hours of life and treatment with TH.

**Methods:**

A systematic review was performed. Inclusion criteria were studies including data of neonates with HIE, treated or not with TH, monitored with aEEG and with neurodevelopmental follow-up of at least 12 months. The period of bibliographic search was until February 2016. No language restrictions were initially applied. Consulted databases were MEDLINE, Scopus, CINHAL and the Spanish language databases GuiaSalud and Bravo. Article selection was performed by two independent reviewers. Quality for each individual paper selected was evaluated using QUADAS-2. Review Manager (RevMan) version 5.3 software was used. Forest plots were constructed to graphically show sensitivity and specificity for all included studies, separating patients treated or not with hypothermia. Summary statistics were estimated using bivariate models and random effects approaches with the R package MADA from summary ROC curves. Meta-regression was used to estimate heterogeneity and trends.

**Results:**

from the 403 articles initially identified, 17 were finally included and critically reviewed. In infants *not treated with hypothermia* the maximum reliability of an abnormal aEEG background to predict death or moderate/severe disability was at 36 hours of life, when a positive post-test probability of 97.90% was achieved (95%CI 88.40 to 99.40%). Positive likelihood ratio (+LR) at these hours of life was 26.60 (95%CI 4.40 to 94.90) and negative likelihood ratio (-LR) was 0.23 (95%CI 0.10 to 0.44). A high predictive value was already present at 6 hours of life in this group of patients, with a positive post-test probability of 88.20% (95%CI 79.80 to 93%) and a +LR of 4.34 (95%CI 2.31 to 7.73). In patients *treated with TH* the maximum predictive reliability was achieved at 72 hours of life (post-test probability of 95.70%, 95%CI 84.40 to 98.50%). +LR at this age was 24.30 (95%CI 5.89 to 71.30) and–LR was 0.40 (95%CI 0.25 to 0.57). Predictive value of aEEG at 6 hours of life was low in these patients (59.10%, 95%CI 55.70 to 63%).

**Conclusion:**

This study confirms that aEEG´s background activity, as recorded during the first 72 hours after birth, has a strong predictive value in infants with HIE treated or not with TH. Predictive values of traces throughout the following 72 hours are a helpful guide when considering and counselling parents about the foreseeable long-term neurological outcome

## Introduction

Perinatal hypoxic ischemic encephalopathy (HIE) is an important cause of early mortality and permanent major disability in full term infants [1,2]. Therapeutic hypothermia (TH) remains the only neuroprotective treatment available for these patients today [3,4]. Perinatal management and prognostic value of clinical evaluation and diagnostic tools have changed with the generalization of TH [5–7].

Amplitude-integrated electroencephalography (aEEG) is a simplified bedside neurophysiology tool that is nowadays widely used in neonatal units today [8] In term newborns with HIE, different authors have described a good correlation of aEEG with conventional EEG findings [9–11]. aEEG traces are assesed using two methods: voltage and background pattern [12,13] Both methods seem to be comparable for rapid clasification of electrocortical activity in newborns [14].

Prior to the use of TH for neonates with HIE, abnormal aEEG background pattern during the first 3–6 postnatal hours was reported to be highly predictive of adverse outcome, with the combination of aEEG pattern and clinical examination being more predictive than either of these parameters independently [12,15–16]. With the use of TH some authors described that normal aEEG background pattern continued to relate to good neurological outcome whilst persistance of an abnormal aEEG tracing beyond 48 hours of life related to later altered neurodevelopmental outcome [17]. However, the temporal profile of the predictive ability of the aEEG in infants treated with TH during the first 72 hours of life has not been established [18].

A systematic review of the medical literature was performed to ascertain the prognostic value of aEEG in newborns with HIE considering hours of life and treatment with hypothermia. This review was part of the Spanish National Health System Clinical Practice Guideline (CPG) on HIE [19].

## Methods

We conducted a systematic review and performed a meta-analysis (MA). Inclusion criteria were studies including newborns with HIE, treated or not with hypothermia, monitored with aEEG and with neurodevelopmental follow-up of at least 12 months.

### Data sources and search strategy

Librarians and the CPG´s panel group performed the electronic literature research. Consulted databases were MEDLINE, Scopus, CINHAL and the Spanish language databases GuiaSalud and Bravo. Clinical trials registers were checked for on-going studies. The period of bibliographic search was until February 2016. No language restrictions were initially applied. Bibliographic references of selected papers were tracked if pertinent. A combination of the following subject headings and keywords was adapted for each electronic database: “Hypoxic ischemic encephalopathy”, “hypoxia-ischemia”, “brain”, “anoxia”, “brain injuries”, “hypoxia brain”, “brain ischemia”, “asphyxia”, “newborn”, “developmental disabilities”, “neurodevelopment”, “cerebral palsy”, “infant mortality”, “hypothermia”, “hypothermia induced”, “neurological morbidity”, “cerebral damage”, “severe hypoxia”, “asphyxia neonatorum”, “oxygen inhalation therapy”, “resuscitation”, “fatal outcome”, “treatment outcome”, “seizures” and “electroencephalography”. Initial search strategy used in Pubmed is shown in S1 Fig. PRISMA checklist is shown in S2 Fig.

### Study selection and data extraction

Article selection was performed by two independent reviewers (DB and RR) and in case of discordance a third author intervened (AGA). Only studies including: 1) human infants with a gestational age of 35 weeks or more, 2) neonatal encephalopathy caused by perinatal asphyxia and 3) neurodevelopmental outcome data available at a postnatal age of at least 12 months were eligible. Review articles, opinion articles, and editorials were excluded as well as abstracts and conference proceedings. A standardized extraction form was used to record the characteristics of each study. For each paper aEEG background pattern at 6, 24, 26, 48 and 72 hours of life was analysed and neurodevelopmental outcome after follow up was recorded. Rates of true positive, true negative, false positive and false negative were recorded. aEEG background trace was considered abnormal according to the pattern classification if the trace was flat, low voltage or burst suppression [13]. Using the voltage classification, aEEG was considered abnormal if the trace’s lower margin was below 5uV and its upper margin was below 10uV [12]. Adverse neurological outcome was defined as the combination of death or moderate/severe disability.

### Quality assessment

Quality for each individual paper selected was evaluated using QUADAS-2 [20]. Quality was considered high if included population and outcomes were well defined. Quality was lowered if population was not well defined, follow-up was irregular or outcomes were not well specified. Influence of aEEG´s result on withdrawal of life support was not specifically described by any of the included articles except for the paper by Eken et al [21].

### Statistical methods

Using Review Manager (RevMan) version 5.3 software, forest plots were constructed to graphically show sensitivity and specificity for all included studies, separating patients receving TH from patients not receiving this therapy. Summary statistics (sensitivity, specificity, likelihood ratios, diagnostic odds ratio) were estimated using bivariate models and random effects approaches with the R package MADA, from the summary receiver operative operating characteristic curve (SROC). MADA uses a linear mixed model with known variances of the random effects, similar to the computational approach by Reitsma et al [22]. Heterogeneity and trends were explored using meta-regression with the same package (S1 Table). Significant heterogeneity at 6 and 24 hours of life, depending on hypothermia treatment led us to separate summary estimates for patients receiving and not receiving hypothermia treatment. Post-test probabilities were computed for different hours of life, using our likelihood ratio estimations. For pre-test probabilities, the point estimations of incidences of death and moderate/severe disability reported by Tagin et al [3] were used.

## Results

**Fig 1:**
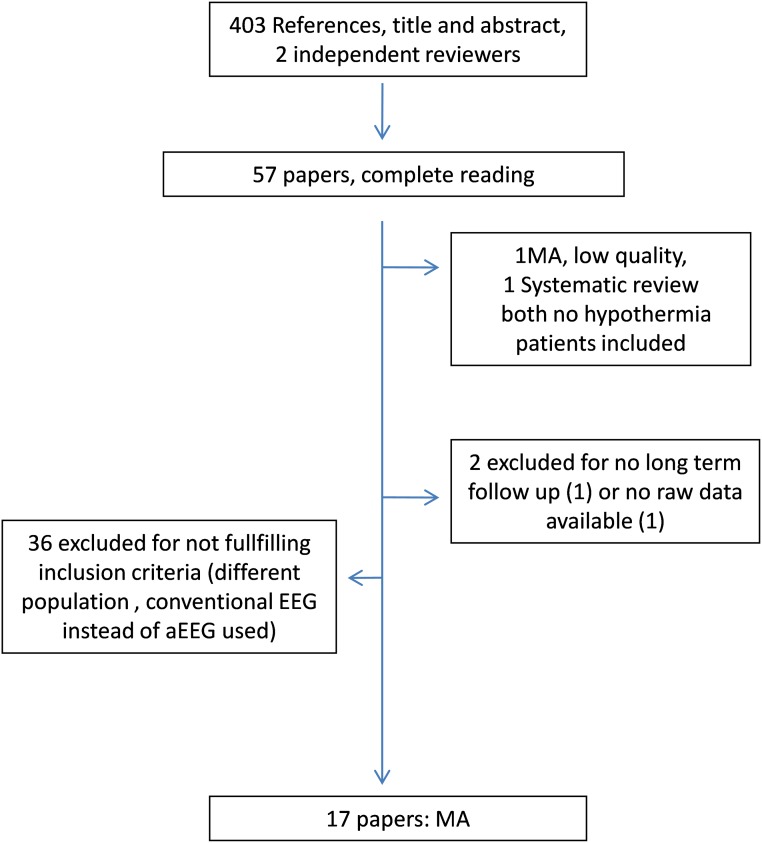
Search and section process flowchart.

**Fig 2:**
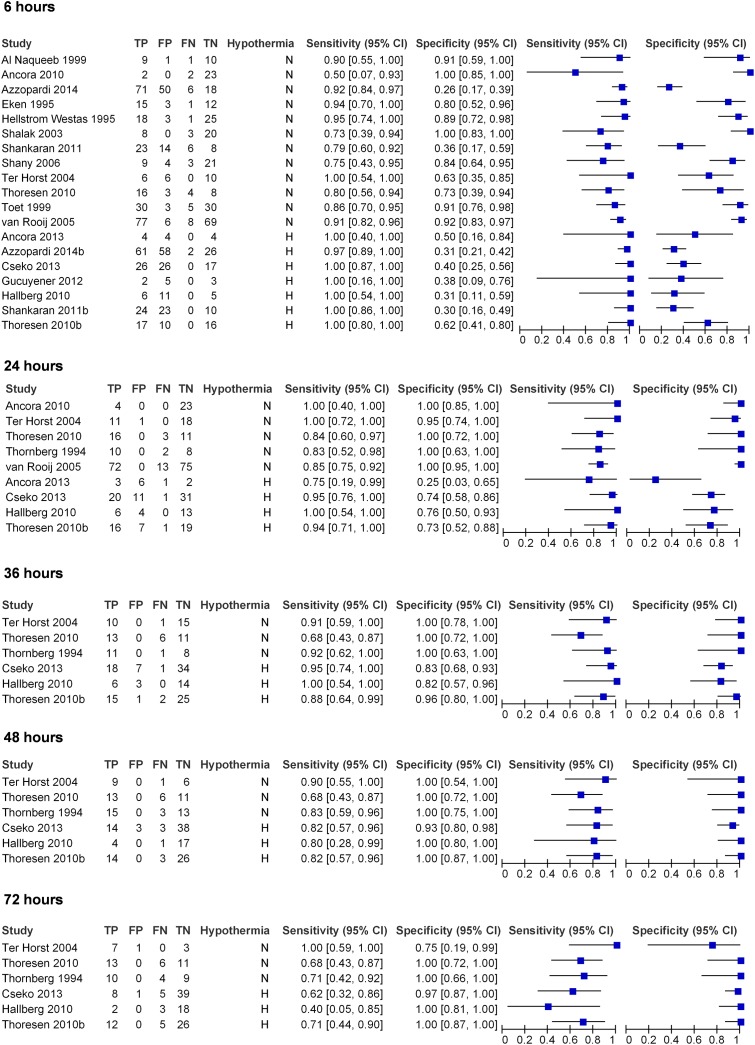
Forest plot for all included studies, considering hours of life and treatment or not with hypothermia.

Four hundred and three articles were identified. Fifty-seven publications were initially preselected on the basis of the article´s title and abstract. Finally, only 17 articles met the inclusion and exclusion criteria (Fig 1) and were critically reviewed. Tables 1 and 2 present the characteristics of all included articles [12,15–17,21,23–34]. HIE stage was graded according to criteria by Sarnat [35]. QUADAS-2 evaluation for these papers is shown in Fig 2. Excluded full text papers and reasons for exclusion are shown in S2 Table.

**Table 1:** Characteristics of included articles with no hypothermia treatment.

Study and year of publication	HIE stage 1/2/3 (n)	NT	Follow-up	Neurodevelopmenta assessment. Definition of adverse outcome	Quality; Reason for reduction
**Thornberg1994** [24]	NS, total n = 38	38	18 mo	Denver; Death or CP ± mental retardation	Moderate; Small sample size, stage of encephalopathy NS, variable follow-up
**Hellström-Westas 1995** [23]	NS, total n = 47, stage 2–3 n = 24, unspecified number of non-encephalopathic infants	47	12–18 mo	NS; Death, CP or psychomotor retardation requiring special training	Moderate; Small sample size, patients with no encephalopathy or stage 1 included, unprecise definition of neurological impairment
**Eken 1995** [21]	11/7/16	34	24 mo	Griffiths and neurologic examination according to Amiel-Tison, Grenier and Touwen.; Death,,DQ <85 or CP	Moderate; Small sample size, patients with HIE stage 1 included
**al Naqeeb 1999** [12]	14/17/7, stage NA in 2	40	18–24 mo	Griffiths or Optimality Score and neurologic examination; Death, neuromotor abnormalities, Optimality Score <20 or DQ <85	Moderate; Small sample size, patients with HIE stage 1 included
**Toet 1999** [15]	NS, total n = 68	68	≥12 mo	Griffiths and neurologic examination according to Amiel-Tison, Grenier and Touwen. Alberta Infant Motor Scale in children younger than 18 mo.; Death, CP or DQ <80	Moderate; Stage of encephalopathy NS, variable follow-up
**Shalak 2003** [16]	3/17/2, normal n = 15, stage 1–2 n = 13	50	18 mo	Bayley, neurological examination, visual and hearing evaluation.; Death, CP, non-specified low Bayley score, altered vision or hearing	Low; Small sample size, patients with no encephalopathy or stage 1 included, disability not well defined
**Ter Horst 2004** [25]	4/18/5, stage NA in 3	30	24 mo	Neurologic examination based on Touwen.; Death, severe mental and motor delay, infantile spasms or CP	Low; Retrospective, small sample size, patients with HIE stage 1 included
**Van Rooij 2005** [26]	NS, total n = 160	160	24 mo	Griffiths; Death, CP, DQ <85	Moderate; Retrospective, stage of encephalopathy NS, variable follow-up
**Shany 2006** [27]	16/14/9	39	30 mo	Bayley II for children ≤42 mo and Leiter-R test for children > 42 mo. Neurologic evaluation according to Amiel-Tison; Death, developmental score <80 or CP	Low; Retrospective, developmental results needing adjustment for maternal education, patients with HIE stage 1 included
**Ancora 2010** [28]	17/13/2	32	24 mo	Griffiths, neurologic examination and psychomotor assessment via Milani-Comparetti’s neuroevolutive assessment, Brazelton’s behavioural assessment and Prechtl’s general movement assessment. Brainstem auditory evoked responses and assessment of visual function.; Death, CP, DQ <85, hearing loss or cortical visual impairment	Moderate; Small sample size, patients with HIE stage 1 included

CP, cerebral palsy; DQ, developmental quotient; NA, not assessable; NS, not specified; NT, cases treated in normothermia; mo = months

**Table 2:** Characteristics of included articles with hypothermia treatment.

Study and year of publication	HIE stage 1/2/3^a^ (n)	NT/HT (n)	Follow-up	Neurodevelopmental assessment; Definition of adverse outcome	Quality; Reason for reduction
**Hallberg 2010** [29]	3/16/4	0/23	12 mo	Serial neurological examinations and AIMS at 4 mo; Death or spasticity and AIMS score <5^th^ percentile	Moderate; Small sample size, retrospective, patients with HIE stage 1 included, short and limited follow-up
**Thoresen 2010** [17]	NS, total n = 74, all stage 2 or 3	31/43	18 mo	Bayley II, neurological examination, GMFCS and visual assessment; Death, MDI <70, GMFCS level 3–5 or no useful vision	High
**Ancora 2013** [30]	0/8/4	0/12	≥12 mo	Griffiths; Death, CP or DQ <88.7	Low; Small sample size, variable follow-up
**Shankaran 2011** [31]	0/71/37	51/57	18 mo	Bayley II, neurological examination, GMFCS, hearing and visual assessment; Death; MDI 70–84 and either a GMFCS level of 2, hearing impairment with no amplification or persistent seizure disorder; MDI <70; GMFCS level 3–5; hearing impairment requiring hearing aids or blindness	High
**Gucuyener 2012** [32]	0/6/4	0/10	8–24 mo	Bayley II; Death, CP, MDI or PDI <70	Low; Retrospective, inconsistent follow-up
**Csekö 2013** [33]	NS, total n = 70, all stage 2 or 3	0/70	18–24 mo	Bayley II; Death, MDI or PDI <70	Moderate; Retrospective
**Azzopardi 2014** [34]	NS, total n = 314, all stage 2 or 3	158/156	18 mo	Bayley II, neurological examination, GMFCS and visual assessmentDeath, CP with GMFCS level 3–5, MDI <70 or bilateral cortical visual impairment	High

AIMS, Alberta Infant Motor Scale; CP, cerebral palsy; DQ, developmental quotient; GMFCS, Gross Motor Function Classification System; MDI, mental developmental index; NT/HT, ratio of cases treated in normothermia/receiving therapeutic hypothermia; PDI, psychomotor developmental index; mo, months

**Fig 3:**
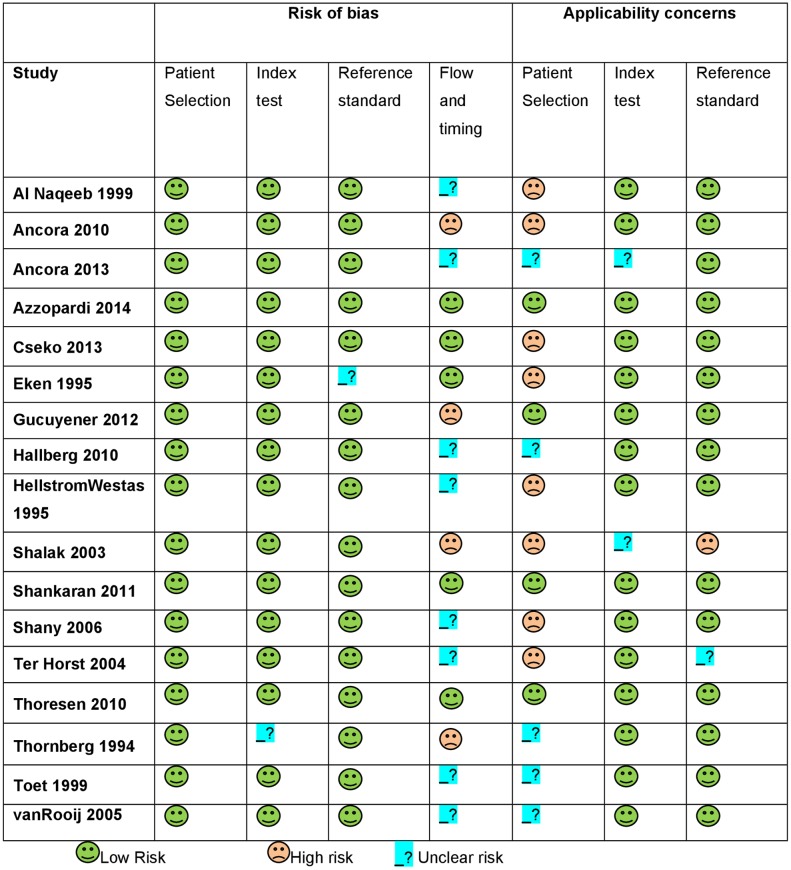
QUADAS 2 assessment of included papers.

Forest plot of sensitivity and specificity with 95% confidence intervals is shown in Fig 3. To evaluate the predictive value of an abnormal aEEG in infants *not treated with hypothermia* thirteen studies including 671 patients were included [12,15–17,21,23–28,31,34]. Seven studies including 360 patients assessed the predictive value of an abnormal aEEG in patients *treated with hypothermia* [17,29–34].

**Fig 4:**
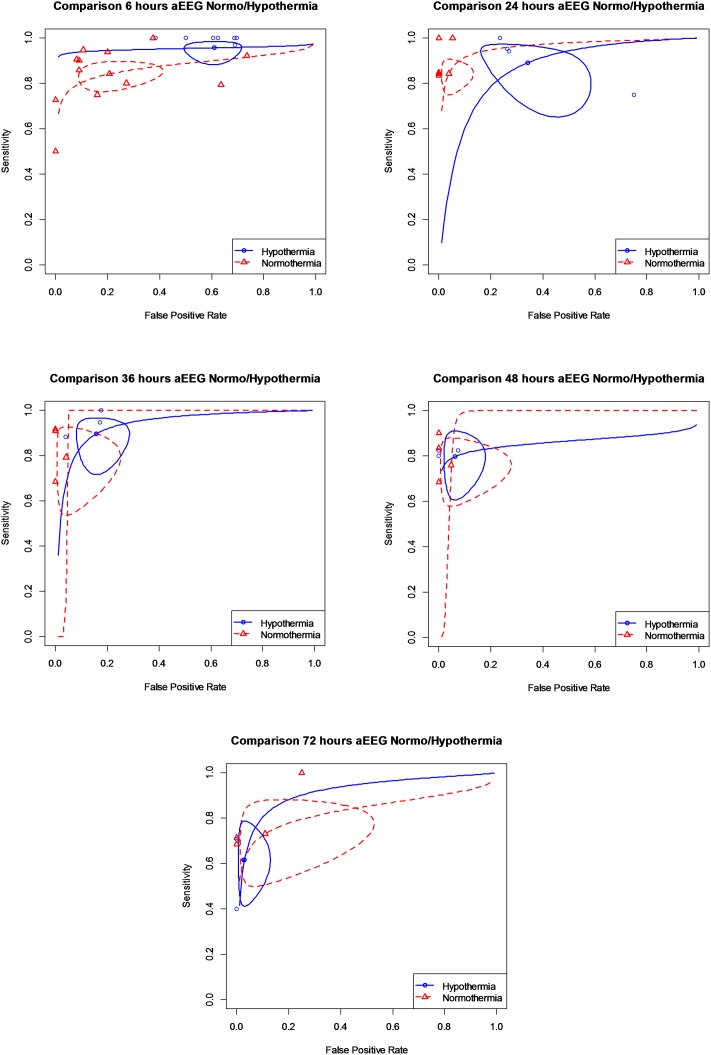
ROC curves for different hours of life.

**Fig 5:**
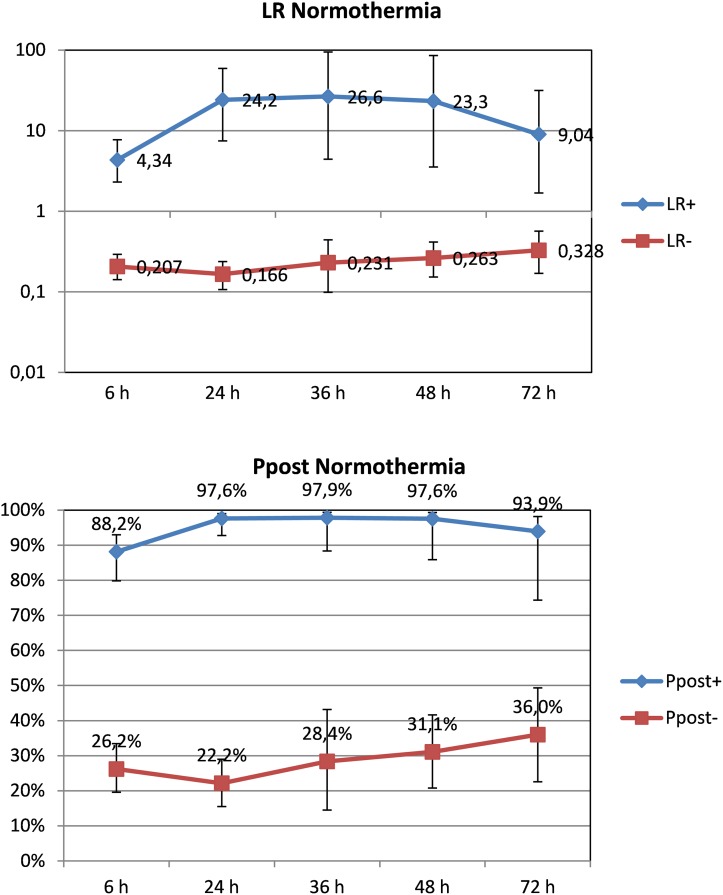
Likelihood ratios and post-test probability of abnormal aEEG and adverse neurological outcome, patients not treated with hypothermia according to hours of life.

ROC curves for different times of life for patients receiving or not TH are shown in Fig 4. Curves for patients receiving and not receiving TH are presented separately, as the prognostic value of aEEG differed for these two groups of patients at 6 and 24 hours of life. A statistical significant trend exists for sensitivity and specificity with increasing hours of life in patients receiving TH. This trend exists only for sensitivity in normothermia patients. After 36 hours of life, ROC curves for patients receiving and not receiving TH treatment overlapped. Pooled sensitivity and specificity, diagnostic odds ratios and an overall summary of this review are given in Table 3. In infants *not treated with hypothermia* the maximum reliability of an abnormal aEEG background to predict death or moderate/severe disability was at 36 hours of life, when a positive post-test probability of 97.90% (95%CI 88.40 to 99.40%) was achieved (Fig 5). Positive likelihood ratio at these hours of life was 26.60 (95%CI 4.40 to 94.90) and negative likelihood ratio 0.23 (95%CI 0.10 to 0.44). Positive post-test probability was already very high at 24 hours of life (97.60%, 95%CI 92.80% to 99%). In the following time epochs, aEEG´s predictive reliability remained high at around 95%. Notably, a high predictive value was already present at 6 hours of life in this group of patients, with a positive post-test probability of 88.20% (95%CI 79.80 to 93%) and a positive likelihood ratio of 4.34 (95%CI 2.31 to 7.73).

**Table 3:** Pooled sensitivity and specificity, diagnostic odds ratio and likelihood ratios with confidence intervals for abnormal aEEG and death or moderate/severe disability, patients receiving and not receiving TH.

aEEG-Death/Seriousdisability		Normothermia	Hypothermia
**6h.**	**Studies (n)**	12 (671)	7 (360)
	**AUC**	0.87	0.94
	**Sensitivity (95%CI)**	0.84 (0.78 to 0.88)	0.95 (0.9 to 0.98)
	**Specificity (95%CI)**	0.79 (0.63 to 0.89)	0.61 (0.51 to 0.69)
	**Positive LR (95%CI)**	4.34 (2.31 to 7.73)	1.57 (1.37 to 1.85)
	**Negative LR (95%CI)**	0.2 (0.14 to 0.29)	0.12 (0.05 to 0.25)
	**DOR (95%CI)**	22.2 (8.85 to 46.2)	15.3 (5.5 to 32.4)
**24h.**	**Studies (n)**	5 (267)	4 (141)
	**AUC**	0.95	0.86
	**Sensitivity (95%CI)**	0.84 (0.77 to 0.89)	0.89 (0.71 to 0.96)
	**Specificity (95%CI)**	0.96 (0.89 to 0.98)	0.65 (0.46 to 0.81)
	**Positive LR (95%CI)**	24.2 (7.48 to 59.3)	2.76 (1.48 to 5.27)
	**Negative LR (95%CI)**	0.16 (0.1 to 0.23)	0.19 (0.04 to 0.53)
	**DOR (95%CI)**	155 (41.6 to 451)	23.9 (2.65 to 95.7)
**36h.**	**Studies (n)**	3 (76)	3 (126)
	**AUC**	0.95	0.93
	**Sensitivity (95%CI)**	0.79 (0.59 to 0.9)	0.89 (0.76 to 0.95)
	**Specificity (95%CI)**	0.95 (0.81 to 0.99)	0.84 (0.74 to 0.9)
	**Positive LR (95%CI)**	26.6 (4.43 to 94.9)	5.73 (3.35 to 9.43)
	**Negative LR (95%CI)**	0.23 (0.09 to 0.44)	0.14 (0.05 to 0.28)
	**DOR (95%CI)**	141 (13.6 to 589)	51.2 (13.2 to 130)
**48h.**	**Studies (n)**	3 (77)	3 (123)
	**AUC**	0.95	0.85
	**Sensitivity (95%CI)**	0.76 (0.61 to 0.86)	0.79 (0.64 to 0.89)
	**Specificity (95%CI)**	0.95 (0.79 to 0.99)	0.93 (0.85 to 0.97)
	**Positive LR (95%CI)**	23.3 (3.55 to 85.7)	14.4 (5.51 to 32.5)
	**Negative LR (95%CI)**	0.26 (0.15 to 0.41)	0.22 (0.11 to 0.37)
	**DOR (95%CI)**	101 (11.3 to 385)	73.7 (17.2 to 200)
**72h.**	**Studies (n)**	3 (64)	3 (119)
	**AUC**	0.82	0.91
	**Sensitivity (95%CI)**	0.73 (0.54 to 0.85)	0.61 (0.45 to 0.75)
	**Specificity (95%CI)**	0.89 (0.58 to 0.97)	0.97 (0.9 to 0.99)
	**Positive LR (95%CI)**	9.04 (1.69 to 31.6)	24.3 (5.89 to 71.3)
	**Negative LR (95%CI)**	0.32 (0.17 to 0.56)	0.39 (0.25 to 0.57)
	**DOR (95%CI)**	31.8 (3.36 to 119)	65.6 (12.7 to 203)

AUC: area under the curve; DOR diagnostic odds ratio; LR likelihood ratio

**Fig 6:**
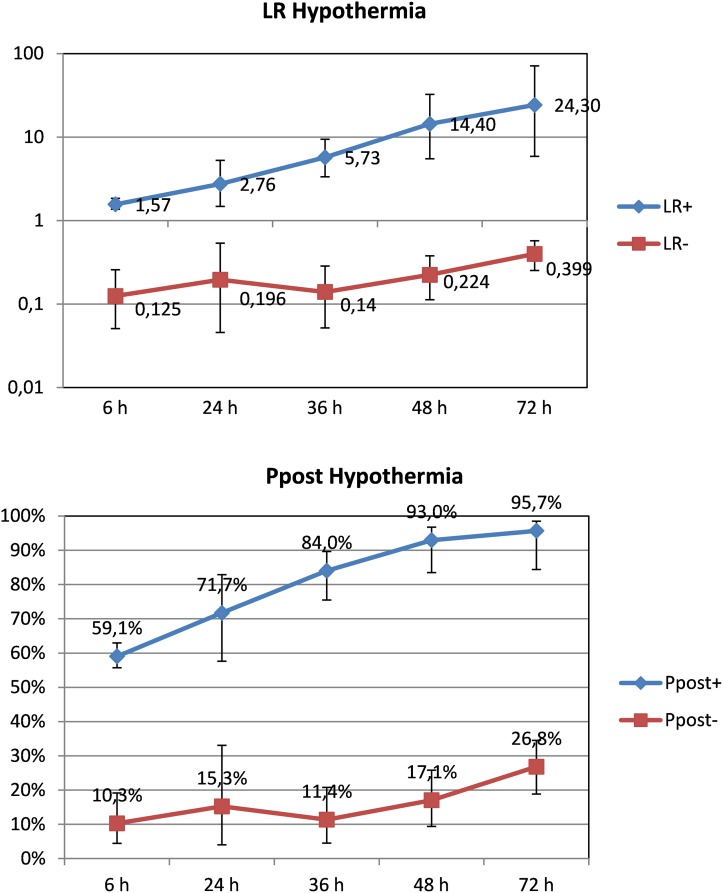
Likelihood ratios and post-test probability of abnormal aEEG and adverse neurological outcome, patients treated with hypothermia according to hours of life.

The maximum predictive reliability for death or moderate/severe disability of an abnormal aEEG in patients *treated with hypothermia* was achieved at 72 hours of life (post-test probability of 95.70%, 95%CI 84.40 to 98.50%). Positive likelihood ratio at this age was 24.30 (95%CI 5.89 to 71.30) and negative likelihood ratio 0.40 (95%CI 0.25 to 0.57). Compared with newborns not treated with TH, the predictive value of aEEG at 6 hours of life (Fig 6) was low in these patients (59.10%, 95%CI 55.70 to 63%).

## Discussion

This systematic review provides a comprehensive summary of the prognostic value of aEEG in newborns with HIE. It is the first review that includes infants treated with hypothermia and, more importantly, stratifies the results according to postnatal age. This study confirms that aEEG background activity, as recorded during the first 72 hours after birth, has a strong predictive value in infants with HIE treated or not with TH.

In 2007 Spitzmiller et al [18] published a MA of the prognostic value of aEEG in newborns with HIE. Papers included were published between 1996 and 2005. In this MA, an abnormal aEEG had high positive and negative likelihood ratios for the prediction of death or moderate to severe disability (+LR 10.1, 95%CI 5.5 to 18; -LR 0.09, 95%CI 0.06 to 15). Limitations of this MA were heterogeneity between studies and different times of aEEG pattern assessment. Also no studies with hypothermia were included, as the analysis was performed in the pre-hypothermia era. Van Laerhoven et al recently reviewed the different prognostic tools for newborns with HIE [36]. These authors presented their results for aEEG as pooled sensitivities and specificities at 6, 24 and 72 hours of life. During the first 6 hours of life, pooled sensitivity for the prediction of adverse outcome was of 0.95 (95%CI 0.87 to 0.98) and pooled specificity was of 0.92 (95%CI 0.61 to 0.99). At 72 hours of life, pooled sensitivity was 0.92 (95%CI 0.66 to 0.99) and pooled specificity was 0.83 (95%CI 0.64 to 0.93). Though the authors conclude that their results could be used in infants treated with hypothermia, no hypothermia studies were included in their review.

Determining which infants with HIE will have significant brain damage is crucial. Among the diverse tools that can assist with prognosis are clinical examination, electrophysiological investigations including aEEG or imaging of the brain. Treatment with hypothermia has indeed changed the prognostic value of the initial aEEG trace and the time cutoffs for outcome prediction based on aEEG in newborn infants with HIE. The seminal work by Thoresen et al [17] showed that the positive predictive value of aEEG before 6 hours of life was significant in normothermia-treated neonates following perinatal HIE (84%), but lower among hypothermia-treated infants (59%). Our analysis showed similar predictive values, with clear differences existing between neonates treated or not with TH. As Infants with moderate or severe HIE are cooled within the first 6 hours of life, differences in aEEG´s predictive values at this early age deserves an explanation. Although profound hypothermia depresses EEG voltages, it has been shown that moderate cooling has no effect on aEEG background pattern both in animal models [37] and clinical studies [38]. Reduction in the predictive reliability of early aEEG (6 and 24 hours of life) for adverse outcome in hypothermia-treated infants compared with normothermic infants could be explained by the beneficial effect of TH on the ongoing brain damage [3]. Additional neuroprotection due to sedative drugs frequently administered in patients receiving hypothermia cannot be ruled out [39]. Another important finding in in the work by Thoresen [17] was that whilst all infants cared for at normothermia who normalized their background pattern beyond 24 hours after birth had a poor outcome, some of the hypothermia-treated infants still developed normally after an abnormal aEEG at 24 hours as long as the aEEG recovered before 48 hours. Our study confirmed a similarly high predictive reliability for death or moderate/severe disability of an abnormal aEEG at 48 hours of life in patients treated with hypothermia (post-test probability of 93%, 95%CI 83.5 to 96.8%).

The effect of TH on tools for neurological assessment of the newborn with HIE other than aEEG has been evaluated in individual studies. In a similar way to aEEG, the predictive value of the severity of clinical encephalopathy at less than 6 hours of age is lower for hypothermia-treated than for normothermia-treated infants [40, 41]. The time course of clinical encephalopathy throughout TH is valuable in outcome prediction. While TH reduces brain injury on magnetic resonance imaging (MRI) in newborn infants with HIE, the predictive value of MRI for subsequent outcome is not affected by TH [42]. Combined with clinical and neuroimaging information, aEEG is of valuable help to the clinician when considering the need for neuroprotection and the likely outcome of infants with HIE.

The strength of the present work is the differentiation of prognostic values depending on postnatal age and use or not of TH. Traces at less than 6 hours and in normothermia conditions are useful for the selection of infants with significant HIE needing neuroprotection in order to improve outcome. Predictive values of traces throughout the following 72 hours are a helpful guide when considering and counselling parents about the foreseeable long-term neurological outcome. In order to provide strong evidence of the predictability of aEEG and its 72-hour time course in infants with perinatal HIE a thorough revision of available literature was needed.

We are conscious of several limitations of our work. The aim to include evaluation at different epochs of postnatal life lowered the number of patients included for each period. Also, heterogeneity was high when considering timing and duration of aEEG monitoring, duration of follow-up and neurodevelopmental tests used by different authors. In studies without TH the percentage of patients with mild HIE is higher than in studies using TH, a factor that might influence aEEG´s prognostic reliability. In addition, the ability of different recording equipment used from initial studies to the latest may be different. Our analysis did not include sleep-wake cycling, seizures or administration of anticonvulsants or narcotics. One important limitation is the general lack of information in the original papers on the numbers of deaths that occurred after withdrawal of intensive care as a consequence of abnormal results of prognostic tools, including aEEG. Of the studies included, only one from the era preceding TH by Eken gave account of the causes of death [21]. In this study, 9 of 17 deaths followed withdrawal of intensive care occurring as a result of a flat aEEG trace beyond 24 hours of life. This limitation could introduce a bias in the prognostic reliability of aEEG.

## Conclusion

This new MA of the prognostic value of aEEG in newborns with HIE incorporates for the first time hours of life and treatment with hypothermia. With TH the maximum capacity reliability of aEEG to predict adverse neurological outcome is delayed from 24–36 to 48–72 hours of life.

## Supporting Information

S1 Fig Initial search strategy used in PubMed. (DOCX)

S2 Fig PRISMA checklist. (DOC)

S1 Table Meta-regression including hours of life and treatment with hypothermia. (DOCX)

S2 Table List of full-text excluded articles and reasons for exclusion. (DOCX)
