# Supplementary material for: Amplitude Integrated Electroencephalogram as a Prognostic Tool in Neonates with Hypoxic-Ischemic Encephalopathy: A Systematic Review
Source: PLoS One. 2016 Nov 1;11(11):e0165744. doi: 10.1371/journal.pone.0165744 (PMC5089691; doi:10.1371/journal.pone.0165744)
Supplement: S1 Fig — (DOCX) [file pone.0165744.s001.docx]

**S1 Fig. Initial search strategy used in PubMed**

((((hypoxia ischemia)) OR ("Hypoxia-Ischemia, Brain"[Mesh])) OR (hypoxi* AND ischemi*[ti] OR hypoxi* AND ischemi*[All Fields]))) AND ("Electroencephalography"[Mesh]) AND ((infant[MeSH] OR infant, newborn[MeSH]))

*Límits: newborn, infant,*

*403 papers retrieved (updated March 2016)*

(((((((hypoxia ischemia)) OR ("Hypoxia-Ischemia, Brain"[Mesh])) OR (hypoxi* AND ischemi*[ti] OR hypoxi* AND ischemi*[All Fields]))) AND ("Electroencephalography"[Mesh]) AND ((infant[MeSH] OR infant, newborn[MeSH])))) NOT ("Hypothermia"[Mesh] OR "Hypothermia, Induced"[Mesh]) AND ((infant[MeSH] OR infant, newborn[MeSH]))

*Límits: newborn, infant,*

*294 papers retrieved (updated March 2016)*

(((((((hypoxia ischemia)) OR ("Hypoxia-Ischemia, Brain"[Mesh])) OR (hypoxi* AND ischemi*[ti] OR hypoxi* AND ischemi*[All Fields]))) AND ("Electroencephalography"[Mesh]) AND ((infant[MeSH] OR infant, newborn[MeSH])))) AND ("Hypothermia"[Mesh] OR "Hypothermia, Induced"[Mesh]) AND ((infant[MeSH] OR infant, newborn[MeSH]))

*Límits: newborn, infant,*

*109 papers retrieved (updated March 2016)*
