# Supplementary material for: Amplitude Integrated Electroencephalogram as a Prognostic Tool in Neonates with Hypoxic-Ischemic Encephalopathy: A Systematic Review
Source: PLoS One. 2016 Nov 1;11(11):e0165744. doi: 10.1371/journal.pone.0165744 (PMC5089691; doi:10.1371/journal.pone.0165744)
Supplement: S1 Table — (DOCX) [file pone.0165744.s003.docx]

**S1 Table. Meta-regression including hours of life and treatment with hypothermia.**

| Meta-regression | | Coefficient | SE | p | 95%CI (coefficient) | |
| --- | --- | --- | --- | --- | --- | --- |
| Hypothermia vs Normothermia | |  |  |  |  |  |
| 6 hours | |  |  |  |  |  |
|  | Sensitivity (intercept) | 1.681 | 0.192 | <0.001 | 1.304 | 2.058 |
|  | Sensitivity/hypothermia | 1.399 | 0.501 | **0.005** | 0.416 | 2.381 |
|  | Specificity (intercept) | -1.266 | 0.333 | <0.001 | -1.918 | -0.614 |
|  | Specificity/hypothermia | 1.668 | 0.520 | **0.001** | 0.648 | 2.687 |
| 24 hours | |  |  |  |  |  |
|  | Sensitivity (intercept) | 1.657 | 0.265 | 0.000 | 1.138 | 2.175 |
|  | Sensitivity/hypothermia | 0.449 | 0.557 | 0.421 | -0.644 | 1.541 |
|  | Specificity (intercept) | -3.246 | 0.587 | 0.000 | -4.396 | -2.096 |
|  | Specificity/hypothermia | 2.525 | 0.669 | **<0.001** | 1.214 | 3.836 |
| 36 hours | |  |  |  |  |  |
|  | Sensitivity (intercept) | 1.204 | 0.377 | 0.001 | 0.465 | 1.944 |
|  | Sensitivity/hypothermia | 0.944 | 0.626 | 0.132 | -0.283 | 2.171 |
|  | Specificity (intercept) | -3.137 | 0.835 | <0.001 | -4.772 | -1.501 |
|  | Specificity/hypothermia | 1.461 | 0.890 | 0.101 | -0.284 | 3.206 |
| 48 hours | |  |  |  |  |  |
|  | Sensitivity (intercept) | 1.153 | 0.342 | 0.001 | 0.483 | 1.824 |
|  | Sensitivity/hypothermia | 0.214 | 0.515 | 0.677 | -0.794 | 1.223 |
|  | Specificity (intercept) | -3.004 | 0.838 | <0.001 | -4.645 | -1.362 |
|  | Specificity/hypothermia | 0.287 | 0.970 | 0.767 | -1.613 | 2.188 |
| 72 hours | |  |  |  |  |  |
|  | Sensitivity (intercept) | 0.892 | 0.353 | 0.012 | 0.200 | 1.585 |
|  | Sensitivity/hypothermia | -0.417 | 0.491 | 0.396 | -1.379 | 0.545 |
|  | Specificity (intercept) | -1.891 | 0.706 | 0.007 | -3.276 | -0.507 |
|  | Specificity/hypothermia | -1.589 | 0.955 | 0.096 | -3.461 | 0.282 |
|  |  |  |  |  |  |  |
| Meta-regression | | Coefficient | SE | p | 95%CI (coefficient) | |
| Trend (Hours of life) | |  |  |  |  |  |
| Normothermia | |  |  |  |  |  |
|  | Sensitivity (intercept) | 1835 | 0.170 | <0.001 | 1502 | 2.167 |
|  | Sensitivity/hours | -0.013 | 0.005 | **0.015** | -0.023 | -0.003 |
|  | Specificity (intercept) | -1.469 | 0.411 | <0.001 | -2.275 | -0.664 |
|  | Specificity/hours | -0.024 | 0.014 | 0.085 | -0.052 | 0.003 |
| Hypothermia | |  |  |  |  |  |
|  | Sensitivity (intercept) | 3288 | 0.381 | <0.001 | 2541 | 4.035 |
|  | Sensitivity/hours | -0.039 | 0.008 | **<0.001** | -0.054 | -0.024 |
|  | Specificity (intercept) | 0.847 | 0.187 | <0.001 | 0.481 | 1212 |
|  | Specificity/hours | -0.068 | 0.007 | **<0.001** | -0.082 | -0.053 |
