# Supplementary material for: Amplitude Integrated Electroencephalogram as a Prognostic Tool in Neonates with Hypoxic-Ischemic Encephalopathy: A Systematic Review
Source: PLoS One. 2016 Nov 1;11(11):e0165744. doi: 10.1371/journal.pone.0165744 (PMC5089691; doi:10.1371/journal.pone.0165744)
Supplement: S2 Table — (DOCX) [file pone.0165744.s004.docx]

**S2 Table. List of full-text excluded articles and reasons for exclusion**

|  | **Article reference** |
| --- | --- |
| **Meta-analysis, no HT included** | **1** |
| **Systematic review, no HT studies included** | **2** |
| **Different study population** | **3,5,15,16,27,29** |
| **No long term follow-up included** | **4** |
| **Use conventional EEG** | **6,7,8,9,10,12,13,14,17,18,19,20,21,22,23,25,26,30,31** |
| **Comment to published article** | **11** |
| **General revision article** | **24,28,33,35,38,39,40** |
| **Evaluate Sleep-wake cycles (not aEEG´pattern)** | **32,34** |
| **No raw data available** | **36** |
| **Does not answer research question** | **37** |
|  |  |
|  |  |
|  |  |
|  |  |
|  |  |
|  |  |

1.Spitzmiller RE, Phillips T, Meinzen-Derr J, Hoath SB. [Amplitude-integrated EEG is useful in predicting neurodevelopmental outcome in full-term infants with hypoxic-ischemic encephalopathy: a meta-analysis.](http://www.ncbi.nlm.nih.gov/pubmed/17890403)J Child Neurol. 2007;22(9):1069-78.

*2.*[van Laerhoven H](http://www.ncbi.nlm.nih.gov/pubmed/?term=van%20Laerhoven%20H%5BAuthor%5D&cauthor=true&cauthor_uid=23248219), [de Haan TR](http://www.ncbi.nlm.nih.gov/pubmed/?term=de%20Haan%20TR%5BAuthor%5D&cauthor=true&cauthor_uid=23248219), [Offringa M](http://www.ncbi.nlm.nih.gov/pubmed/?term=Offringa%20M%5BAuthor%5D&cauthor=true&cauthor_uid=23248219), [Post B](http://www.ncbi.nlm.nih.gov/pubmed/?term=Post%20B%5BAuthor%5D&cauthor=true&cauthor_uid=23248219), [van der Lee JH](http://www.ncbi.nlm.nih.gov/pubmed/?term=van%20der%20Lee%20JH%5BAuthor%5D&cauthor=true&cauthor_uid=23248219). Prognostic tests in term neonates with hypoxic-ischemic encephalopathy: a systematic review. [Pediatrics.](http://www.ncbi.nlm.nih.gov/pubmed/?term=van+Laerhoven+2013) 2013;131:88-98.

3.Vasilijevic B, Maglajic-Djukic S, [Gojnić M](http://www.ncbi.nlm.nih.gov/pubmed?term=Gojnić%20M%5BAuthor%5D&cauthor=true&cauthor_uid=22779294). Prognostic value of amplitude-integrated electroencephalography in neonates with hypoxic-ischemic encephalopathy. [Vojnosanit Pregl.](http://www.ncbi.nlm.nih.gov/pubmed/22779294#%23) 2012;69:492-499.

4.Massaro AN, Tsuchida T, Kadom N, El-Dib M, Glass P, Baumgart S [aEEG Evolution during therapeutic hypothermia and prediction of NICU outcome in encephalopathic neonates.](http://www.ncbi.nlm.nih.gov/pubmed/22796967) Neonatology. 2012;102:197-202

# *5.*Jiang C [Yang YH](http://www.ncbi.nlm.nih.gov/pubmed/?term=Yang%20YH%5BAuthor%5D&cauthor=true&cauthor_uid=25727101), [Chen LQ](http://www.ncbi.nlm.nih.gov/pubmed/?term=Chen%20LQ%5BAuthor%5D&cauthor=true&cauthor_uid=25727101), [Shuai XH](http://www.ncbi.nlm.nih.gov/pubmed/?term=Shuai%20XH%5BAuthor%5D&cauthor=true&cauthor_uid=25727101), [Lu H](http://www.ncbi.nlm.nih.gov/pubmed/?term=Lu%20H%5BAuthor%5D&cauthor=true&cauthor_uid=25727101), [Xiang JH](http://www.ncbi.nlm.nih.gov/pubmed/?term=Xiang%20JH%5BAuthor%5D&cauthor=true&cauthor_uid=25727101) et al. Early amplitude-integrated EEG monitoring 6 h after birth predicts long-term neurodevelopment of asphyxiated late preterm infants. Eur J Pediatr 2015;174(8):1043-52.

6.Lamblin MD, Racoussot S , Pierrat V, Duquennoy C, Ouahsine T, Lequien P, et al Encéphalopathie anoxo-ischénique du nouveau-né à terme. Apport de l’électroencephalogramme et de l’échographie transfontanellaire à l’évaluation pronostique. À propos de 29 observations. Neurophysiol Clin. 1996; 26:369-378.

7.Wertheim D, Mercuri E, Faundez JC, Rutherford M, Acolet D, Dubowitz L. [Prognostic value of continuous electroencephalographic recording in full term infants with hypoxic ischaemic encephalopathy.](http://www.ncbi.nlm.nih.gov/pubmed/7979486) Arch Dis Child. 1994;71:F97-F102.

8.Azzopardi D, Guarino I, Brayshaw C, Cowan F, Price-Williams D, Edwards AD, et al. Prediction of neurological outcome after birth asphyxia from early continuous two-channel electroencephalography. Early Hum. Dev. 1999;55:113–123.

*9.* Gire C, Nicaise C, Roussel CM, Soula F, Girard G, Somma-Mauvais, G et al. Encéphalopathie hypixo-ischémique du nouveau-né à terme. Apport de l'électroencéphalogramme et de l'IRM ou de la TDM à l'évaluation pronostique. À propos de 26 observations. Neurophysiol Clin. 2000; 30: 97-107.

10. Biagioni E, Mercuri E, Rutherford M, Cowan F, Azzopardi D, Frisone MF, et al. [Combined use of electroencephalogram and magnetic resonance imaging in full-term neonates with acute encephalopathy.](http://www.ncbi.nlm.nih.gov/pubmed/11230583) Pediatrics. 2001;107:461-468.

*11.* Baumgart S, Graziani LJ. [Predicting the future for term infants experiencing an acute neonatal encephalopathy: electroencephalogram, magnetic resonance imaging, or crystal ball?](http://www.ncbi.nlm.nih.gov/pubmed/11230604). Pediatrics. 2001;107:588-589.

12. Mandel R, Martinot A, Delepoulle F, Lamblin M-D, Laureau E, Vallee L, et al. Prediction of outcome after hypoxic-ischemic encephalopathy: a prospective clinical and electrophysiologic study. J Pediatr. 2002;141:45–50.

13. Leijser LM, Vein AA, Liauw L, Strauss T, Veen S, Wezel-Meijler G. [Prediction of short-term neurological outcome in full-term neonates with hypoxic-ischaemic encephalopathy based on combined use of electroencephalogram and neuro-imaging.](http://www.ncbi.nlm.nih.gov/pubmed/18330835) Neuropediatrics. 2007;38:219-227.

14. Selton D, Andre M. Prognosis of Hipoxic-Ischaemic encephalopathy in full term newborns. Value of neonatal electroencephalography. Neuroped.1997; 28: 276-280.

15. Shany E, Oshra Benzaqen O, and Nathan Watemberg N. Comparison of continuous drip of midazolam or lidocaine in the treatment of intractable neonatal seizures. J Child Neurol. 2007 22: 255-229.

16. [van Rooij LG](http://www.ncbi.nlm.nih.gov/pubmed?term=van%20Rooij%20LG%5BAuthor%5D&cauthor=true&cauthor_uid=17671044), [de Vries LS](http://www.ncbi.nlm.nih.gov/pubmed?term=de%20Vries%20LS%5BAuthor%5D&cauthor=true&cauthor_uid=17671044), [Handryastuti S](http://www.ncbi.nlm.nih.gov/pubmed?term=Handryastuti%20S%5BAuthor%5D&cauthor=true&cauthor_uid=17671044), [Hawani D](http://www.ncbi.nlm.nih.gov/pubmed?term=Hawani%20D%5BAuthor%5D&cauthor=true&cauthor_uid=17671044), [Groenendaal F](http://www.ncbi.nlm.nih.gov/pubmed?term=Groenendaal%20F%5BAuthor%5D&cauthor=true&cauthor_uid=17671044), [van Huffelen AC](http://www.ncbi.nlm.nih.gov/pubmed?term=van%20Huffelen%20AC%5BAuthor%5D&cauthor=true&cauthor_uid=17671044) et al Neurodevelopmental outcome in term infants with status epilepticus detected with amplitude-integrated electroencephalography. [Pediatrics.](http://www.ncbi.nlm.nih.gov/pubmed/17671044#%23) 2007 Aug ; 120:e354-e356.

17. Mariani E, Scelsa B, Pogliani L, Introvini P, Lista G. [Prognostic value of electroencephalograms in asphyxiated newborns treated with hypothermia.](http://www.ncbi.nlm.nih.gov/pubmed/18940554) Pediatr Neurol. 2008 Nov ;39:317-324.

18. Murray DM, Boylan GB, Ryan CA, Connolly S. Early EEG findings in hypoxic ischemic encephalopathy predict outcomes at 2 years. Pediatrics. 2009; 124 (3) : e459-e467.

19. Rafay MF, Cortez MA, de Veber GA, Tan-Dy C, Al-Futaisi A, Yoon W, et al [Predictive value of clinical and EEG features in the diagnosis of stroke and hypoxic ischemic encephalopathy in neonates with seizures.](http://www.ncbi.nlm.nih.gov/pubmed/19478219) Stroke. 2009 Jul ;40: 2402-2407.

20. [Doyle OM](http://www.ncbi.nlm.nih.gov/pubmed?term=Doyle%20OM%5BAuthor%5D&cauthor=true&cauthor_uid=21096334), [Temko A](http://www.ncbi.nlm.nih.gov/pubmed?term=Temko%20A%5BAuthor%5D&cauthor=true&cauthor_uid=21096334), [Murray DM](http://www.ncbi.nlm.nih.gov/pubmed?term=Murray%20DM%5BAuthor%5D&cauthor=true&cauthor_uid=21096334), [Lightbody G](http://www.ncbi.nlm.nih.gov/pubmed?term=Lightbody%20G%5BAuthor%5D&cauthor=true&cauthor_uid=21096334), [Marnane W](http://www.ncbi.nlm.nih.gov/pubmed?term=Marnane%20W%5BAuthor%5D&cauthor=true&cauthor_uid=21096334), [Boylan GB](http://www.ncbi.nlm.nih.gov/pubmed?term=Boylan%20GB%5BAuthor%5D&cauthor=true&cauthor_uid=21096334). Predicting the neurodevelopmental outcome in newborns with hypoxic-ischaemic injury. [Conf Proc IEEE Eng Med Biol Soc.](http://www.ncbi.nlm.nih.gov/pubmed?term=doyle%20hypoxic%20ischemic%20injury%202010#%23) 2010;2010:1370-1373.

21. Hathi M, Sherman DL, Inder T, Rothman NS, Natarajan M, Niesen C, et al. Quantitative EEG in babies at risk for hypoxic ischemic encephalopathy after perinatal asphyxia. J Perinatol. 2010;30:122–126.

22. Flisberg A, Kjellmer I, Löfhede J, Lindecrantz K, Thordstein M. [Prognostic capacity of automated quantification of suppression time in the EEG of post-asphyctic full-term neonates.](http://www.ncbi.nlm.nih.gov/pubmed/21615787) Acta Paediatr. 2011;100:1338-1343.

23. Hamelina S, Delnardb N, Cneudeb F, Debillonb T, Vercueila L. Influence of hypothermia on the prognostic value of early EEG in full-term neonates with hypoxic ischemic encephalopathy. Neurophysiol Clin. 2011; 41: 19-27.

24. Lamblin MD, Andréb M. Électroencéphalogramme du nouveau-né à terme. Aspects normaux et encephalopathy hypoxo-ischémique. Neurophysiol Clin. 2011; 41: 1-18.

25. Nash KB, Bonifacio SL, Glass HC, Sullivan JE, Barkovich AJ, Ferriero DM, et al. Video-EEG monitoring in newborns with hypoxic-ischemic encephalopathy treated with hypothermia. Neurology. 2011;76:556–562

*26.* Wusthoff CJ, Dlugos DJ, Gutierrez-Colina A, Wang A, Cook N, et al. Electrographic seizures during therapeutic hypothermia for neonatal hypoxic-ischemic encephalopathy. J Child Neurol. 2011; 26:724-728.

*27*. vanRooij LG, Toet MC, van Huffelen AC, Groenendaal F, Laan W, et al. Effect of treatment of subclinical neonatal seizures detected with aEEG: randomized, controlled trial. Pediatrics. 2010;125:e358-e366.

28. Hoehn T, Hansmann G, Bührer C, Simbruner G, Gunn AJ, et al. Therapeutic hypothermia in neonates. Review of current clinical data, ILCOR recommendations and suggestions for implementation in neonatal intensive care units. Resuscitation 2008; 78:7-12.

29. Angeles DM, Wycliffe N, Michelson D, Holshouser BA, Deming DD, Pearce WJ, Sowers LC, Ashwal S. Use of opioids in asphyxiated term neonates: effects on neuroimaging and clinical outcome. Pediatr Res. 2005; 57:873-878.

30. Walsh BH, Murray DM, Boylan GB. The use of conventional EEG for the assesment of hypoxic ischaemic encephalopathy in the newborn: a review. Clin Neurophysiol. 2011 Jul ;122:1284-1294.

31. Murray DM, Ryan CA, Boylan GB, Connolly S. Prediction of seizures in asphyxiated neonates: correlation with continuos video-electroencephalographic monitoring. Pediatrics 2006; 116:1001-1006.

32. [Takenouchi T](http://www.ncbi.nlm.nih.gov/pubmed?term=Takenouchi%20T%5BAuthor%5D&cauthor=true&cauthor_uid=21353680), [Rubens EO](http://www.ncbi.nlm.nih.gov/pubmed?term=Rubens%20EO%5BAuthor%5D&cauthor=true&cauthor_uid=21353680), [Yap VL](http://www.ncbi.nlm.nih.gov/pubmed?term=Yap%20VL%5BAuthor%5D&cauthor=true&cauthor_uid=21353680), [Ross G](http://www.ncbi.nlm.nih.gov/pubmed?term=Ross%20G%5BAuthor%5D&cauthor=true&cauthor_uid=21353680), [Engel M](http://www.ncbi.nlm.nih.gov/pubmed?term=Engel%20M%5BAuthor%5D&cauthor=true&cauthor_uid=21353680), [Perlman JM](http://www.ncbi.nlm.nih.gov/pubmed?term=Perlman%20JM%5BAuthor%5D&cauthor=true&cauthor_uid=21353680). Delayed onset of sleep-wake cycling with favorable outcome in hypothermic-treated neonates with encephalopathy.[J Pediatr.](http://www.ncbi.nlm.nih.gov/pubmed?term=Takenouchi%202011%20hypothermia%20sleep#%23) 2011 Aug ;159:232-237.

33.[Sarkar S](http://www.ncbi.nlm.nih.gov/pubmed?term=Sarkar%20S%5BAuthor%5D&cauthor=true&cauthor_uid=18004390), [Barks JD](http://www.ncbi.nlm.nih.gov/pubmed?term=Barks%20JD%5BAuthor%5D&cauthor=true&cauthor_uid=18004390), [Donn SM](http://www.ncbi.nlm.nih.gov/pubmed?term=Donn%20SM%5BAuthor%5D&cauthor=true&cauthor_uid=18004390). Should amplitude-integrated electroencephalography be used to identify infants suitable for hypothermic neuroprotection? [J Perinatol.](http://www.ncbi.nlm.nih.gov/pubmed?term=amplitude%20integrated%20electroencephalography%20Sarkar#%23) 2008 Feb ;28:117-122.

34. [Osredkar D](http://www.ncbi.nlm.nih.gov/pubmed?term=Osredkar%20D%5BAuthor%5D&cauthor=true&cauthor_uid=15687440), [Toet MC](http://www.ncbi.nlm.nih.gov/pubmed?term=Toet%20MC%5BAuthor%5D&cauthor=true&cauthor_uid=15687440), [van Rooij LG](http://www.ncbi.nlm.nih.gov/pubmed?term=van%20Rooij%20LG%5BAuthor%5D&cauthor=true&cauthor_uid=15687440), [van Huffelen AC](http://www.ncbi.nlm.nih.gov/pubmed?term=van%20Huffelen%20AC%5BAuthor%5D&cauthor=true&cauthor_uid=15687440), [Groenendaal F](http://www.ncbi.nlm.nih.gov/pubmed?term=Groenendaal%20F%5BAuthor%5D&cauthor=true&cauthor_uid=15687440), [de Vries LS](http://www.ncbi.nlm.nih.gov/pubmed?term=de%20Vries%20LS%5BAuthor%5D&cauthor=true&cauthor_uid=15687440).Sleep-wake cycling on amplitude-integrated electroencephalography in term newborns with hypoxic-ischemic encephalopathy. [Pediatrics.](http://www.ncbi.nlm.nih.gov/pubmed/15687440#%23) 2005 Feb ;115:327-332.

35. Wyatt JS, Gluckman PD, Liu PY, Azzopardi D, Ballard R, Edwards AD, et al CoolCap Study Group. [Determinants of outcomes after head cooling for neonatal encephalopathy.](http://www.ncbi.nlm.nih.gov/pubmed/17473091) Pediatrics. 2007;119:912-921.

36. [Toet MC](http://www.ncbi.nlm.nih.gov/pubmed?term=Toet%20MC%5BAuthor%5D&cauthor=true&cauthor_uid=16452351), [Lemmers PM](http://www.ncbi.nlm.nih.gov/pubmed?term=Lemmers%20PM%5BAuthor%5D&cauthor=true&cauthor_uid=16452351), [van Schelven LJ](http://www.ncbi.nlm.nih.gov/pubmed?term=van%20Schelven%20LJ%5BAuthor%5D&cauthor=true&cauthor_uid=16452351), [van Bel F](http://www.ncbi.nlm.nih.gov/pubmed?term=van%20Bel%20F%5BAuthor%5D&cauthor=true&cauthor_uid=16452351). Cerebral oxygenation and electrical activity after birth asphyxia: their relation to outcome. Pediatrics; 117:333-339.

# 37. [Marics G](http://www.ncbi.nlm.nih.gov/pubmed/?term=Marics%20G%5BAuthor%5D&cauthor=true&cauthor_uid=24268061) , [Csekő A](http://www.ncbi.nlm.nih.gov/pubmed/?term=Csek%C5%91%20A%5BAuthor%5D&cauthor=true&cauthor_uid=24268061) , [Vásárhelyi B](http://www.ncbi.nlm.nih.gov/pubmed/?term=V%C3%A1s%C3%A1rhelyi%20B%5BAuthor%5D&cauthor=true&cauthor_uid=24268061) , [Zakariás D](http://www.ncbi.nlm.nih.gov/pubmed/?term=Zakari%C3%A1s%20D%5BAuthor%5D&cauthor=true&cauthor_uid=24268061) , [Schuster G](http://www.ncbi.nlm.nih.gov/pubmed/?term=Schuster%20G%5BAuthor%5D&cauthor=true&cauthor_uid=24268061) , [Szabó M](http://www.ncbi.nlm.nih.gov/pubmed/?term=Szab%C3%B3%20M%5BAuthor%5D&cauthor=true&cauthor_uid=24268061). Prevalence and etiology of false normal aEEG recordings in neonatal hypoxic-ischaemic encephalopathy. [BMC Pediatr.](http://www.ncbi.nlm.nih.gov/pubmed/24268061) 2013 22;13:194

# 38. Hellström-Westas. Monitoring brain function with aEEG in term asphyxiated infants before and during cooling. Acta Paediatr. 2013;102(7):678-9.

# *39.* [Sabir H](http://www.ncbi.nlm.nih.gov/pubmed/?term=Sabir%20H%5BAuthor%5D&cauthor=true&cauthor_uid=25457081), [Cowan FM](http://www.ncbi.nlm.nih.gov/pubmed/?term=Cowan%20FM%5BAuthor%5D&cauthor=true&cauthor_uid=25457081). Prediction of outcome methods assessing short- and long-term outcome after therapeutic hypothermia.Semin Fetal Neonatal Med 2015;20(2):115-21.

# 40. [Bonifacio SL](http://www.ncbi.nlm.nih.gov/pubmed/?term=Bonifacio%20SL%5BAuthor%5D&cauthor=true&cauthor_uid=25577654), [deVries LS](http://www.ncbi.nlm.nih.gov/pubmed/?term=deVries%20LS%5BAuthor%5D&cauthor=true&cauthor_uid=25577654), Groenendal F. Impact of hypothermia on predictors of poor outcome: how do we decide to redirect care? [Semin Fetal Neonatal Med.](http://www.ncbi.nlm.nih.gov/pubmed/?term=Impact+of+hypothermia+on+predictors+of+poor+outcome%3A+How+do+we+decide+to+redirect+care%3F) 2015 Apr;20(2):122-7.
